# Accuracy of cup positioning of a non-invasive augmented reality-based navigation system for total hip arthroplasty in the supine position

**DOI:** 10.1186/s42836-025-00327-w

**Published:** 2025-09-01

**Authors:** Ryohei Takada, Naoto Watanabe, Kazumasa Miyatake, Naohiko Sugita, Toshitaka Yoshii, Hideyuki Koga

**Affiliations:** 1 Department of Orthopaedic Surgery, Institute of Science Tokyo Hospital, Tokyo, 113-8519 Japan; 2 Department of Mechanical Engineering, School of Engineering, The University of Tokyo, Tokyo, 113-8656 Japan

**Keywords:** Augmented reality, Navigation system, Total hip arthroplasty, Cup alignment

## Abstract

**Background:**

A new non-invasive augmented reality-based portable navigation system was developed for accurate cup positioning during total hip arthroplasty in the supine position. This study aimed to clarify whether the navigation system supports cup positioning more accurately than a conventional goniometer during surgery.

**Methods:**

In total, 60 patients who underwent total hip arthroplasty in the supine position between September 2021 and August 2022 were retrospectively investigated. The navigation system was used for 30 patients (navigation group), and a conventional goniometer was used for 30 patients (control group) to measure radiographic cup inclination and anteversion during surgery. The primary outcome was the absolute value of the difference in cup alignment measured during surgery and via postoperative radiography.

**Results:**

An assessment of the primary outcome showed no significant difference in the radiographic cup inclination in the navigation and control groups (2.9° vs. 3.2°; mean difference, 0.3°; 95% confidence interval, − 1.4 to 0.9; *P* = 0.67); however, the positioning in the navigation group was significantly more accurate than that in the control group in terms of radiographic anteversion (3.4° vs. 5.4°; mean difference, 2.0°; 95% confidence interval, 0.4–3.8; *P* = 0.017).

**Conclusions:**

A new non-invasive augmented reality-based portable navigation system resulted in more accurate cup anteversion than the conventional goniometer.

## Background

The orientation of the acetabular cup during total hip arthroplasty (THA) is critical for preventing postoperative dislocation, accelerated wear and loosening, reduced range of motion, and patient dissatisfaction [1, 2]. Various computer-assisted devices have been developed worldwide to achieve ideal cup positions, such as computed tomography (CT)-based navigation and other image-free navigation systems, including portable navigation systems [3, 4]. New portable navigation systems using smartphone technology have recently been developed [5]. Smartphones with cameras and augmented reality (AR) technology, allowing for high-quality pictures, can be used to enhance cup positioning accuracy at a lower cost than other navigation systems.

Several concerns regarding these navigation systems have been reported [6–9]. During most navigation system procedures, fixation pins must be inserted in the pelvic crest to trace pelvic movement during surgery; this prolongs surgical duration and is invasive [10]. Other limitations of these navigation systems include patient and surgeon X-ray exposure, high costs, and complicated procedures that prolong surgical duration [11].

This study investigated the accuracy of a new non-invasive AR-based portable navigation system for cup positioning. This system does not need fixation pins to the pelvis. Unlike other navigation systems, the present system acquires the functional pelvic plane (FPP) from the pelvic orientation immediately prior to cup placement [4]. However, the accuracy of the system is still unclear. The research question of this study was: Does a new portable navigation system provide more accurate cup positioning than a conventional goniometer? The hypothesis was that the navigation system would provide more accurate cup alignment than the conventional goniometer.

## Methods

### Patients

**Fig. 1:**
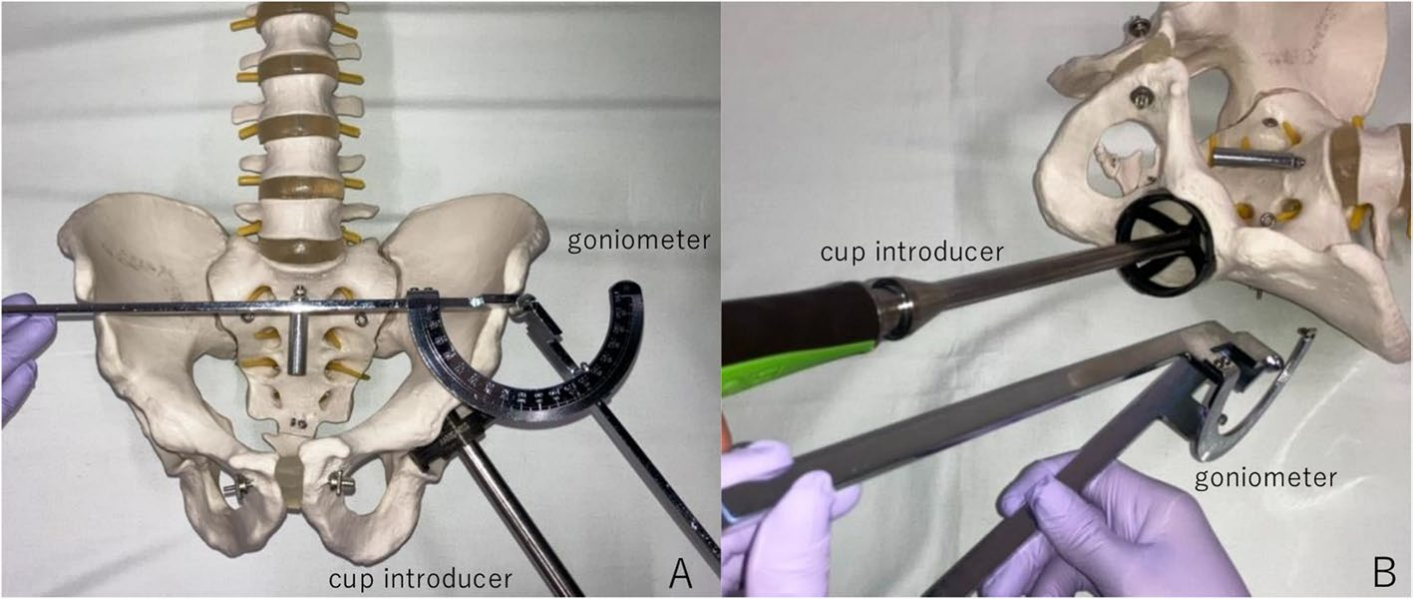
Radiographic inclination and anteversion of the control group measured using a conventional goniometer. **A** The radiographic inclination angle is defined as the angle between the face of the acetabular cup and the transverse axis (a line between the anterior superior iliac spines). **B** Radiographic anteversion is defined as the angle between the acetabular and coronal planes (a surface perpendicular to the direction of gravity)

This study was performed at a single university hospital according to the principles of the Declaration of Helsinki. This research was approved by the institutional research ethics committee, and informed consent was obtained from all patients before surgery. In this study, patients who underwent THA in the supine position between September 2021 and August 2022 were retrospectively investigated. The first 30 cases were performed using a conventional goniometer to decide cup alignment during surgery. The next 30 cases were performed using the navigation system after March 2022, when pharmaceutical approval for this system was obtained. The conventional goniometer was used to measure radiographic cup inclination and anteversion after cup insertion during surgery (Fig. 1).

Patient background and surgical characteristics (age, sex, side, body mass index [BMI], diagnosis, surgical duration, and intraoperative blood loss) were recorded.

All surgeries were performed using a modified Watson–Jones anterolateral approach and Trident II acetabular cups (Stryker, Mahwah, NJ, USA). No additional screws were used for cup fixation. An Accolade II or Exeter stem (Stryker, Mahwah, NJ, USA) was used for all patients. All surgeries were performed under general anesthesia. Three surgeons performed all the surgeries (R.T., N.W., and K.M.).

Femoral preparation was performed first. Subsequently, cup preparation was performed. Cup insertion was performed in a flat, supine position. Femoral preparation was performed in the extended hip position. Radiographic cup inclination was aimed at 40°, and radiographic cup anteversion was at 10–30°. Radiographic cup anteversion was defined intraoperatively, considering femoral stem anteversion [12]. Cup positioning was also adjusted to prevent iliopsoas impingement by ensuring that the anterior edge of the cup did not protrude beyond the anterior wall of the acetabulum [13]. No other assistive devices were used intraoperatively, including radiography and fluoroscopy.

### Non-Invasive AR-Based Navigation

**Fig. 2:**
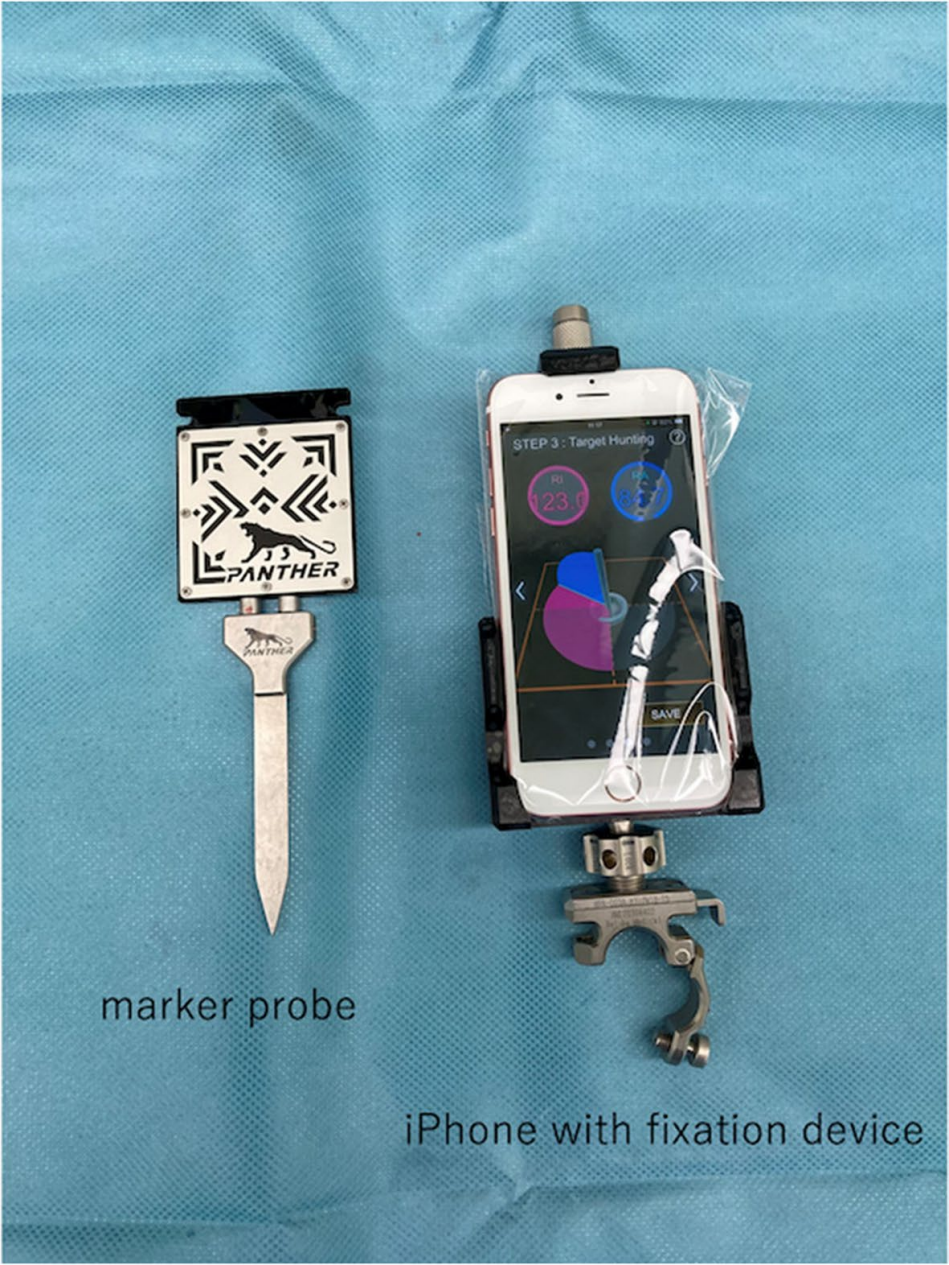
Marker probe and smartphone with a fixation device. The smartphone is contained within a sterile plastic bag

This AR-based navigation system (Ortho Panther; Xelha-Medical Corp, Nagano, Japan) comprises a smartphone application, marker probe, smartphone fixation device, and cup holder (Fig. 2).

This system has been studied and developed in collaboration with the Engineering Department of Tokyo University. Pharmaceutical approval in Japan was obtained in March 2022. Since then, this system has been commercially available only in Japan. The marker probe, fixation device, and cup holder were used after sterilization. The smartphone was placed in a sterilized plastic bag. The smartphone application uses gyro and acceleration sensors to measure smartphone orientation and the gravity vector. The application also uses AR technology to measure marker position, orientation, and probe tip position by capturing marker patterns. The marker pattern and the offset from the marker center to the probe tip are preliminarily programmed and used for the measurement.

**Fig. 3:**
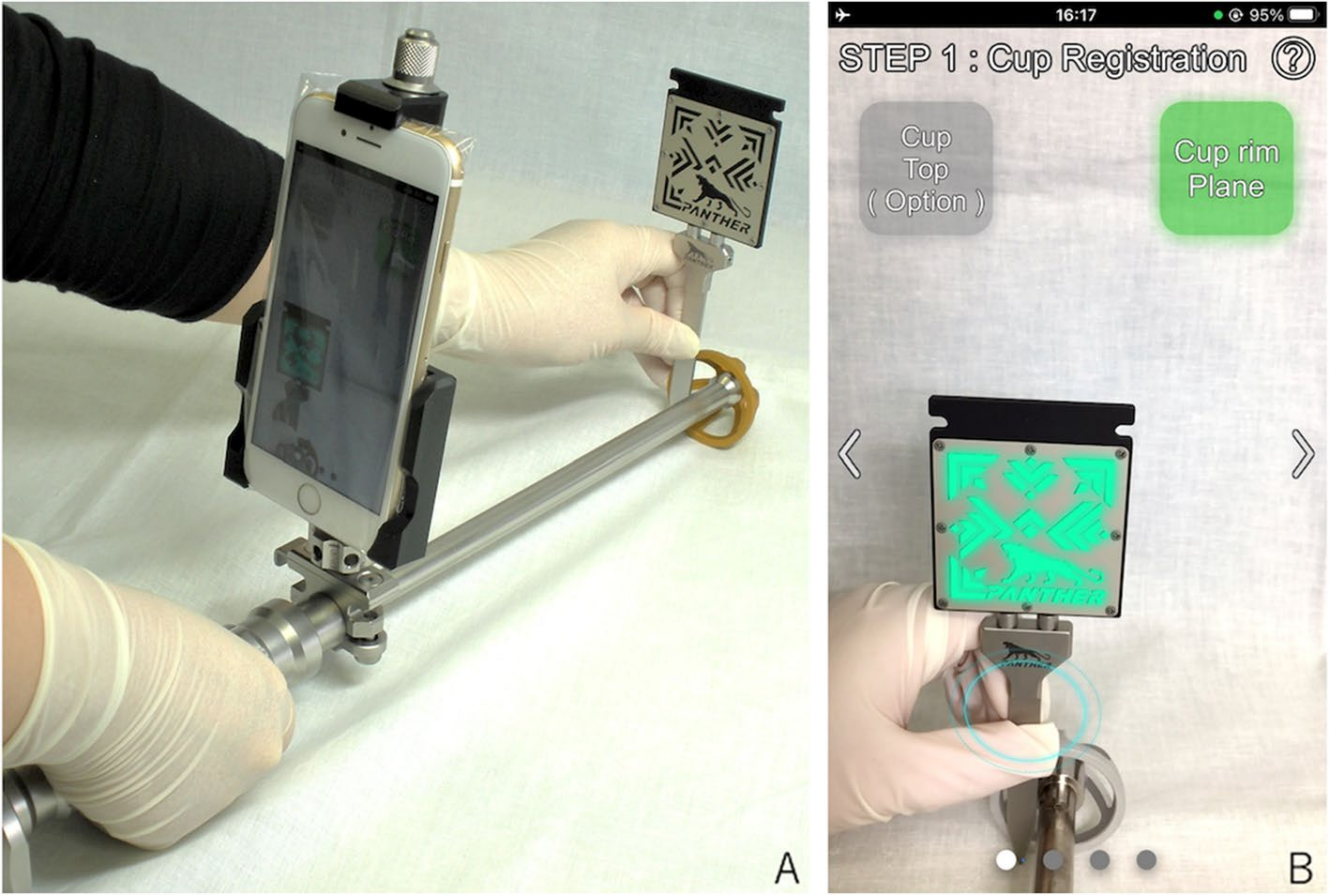
Cup-registration procedure: Step 1. **A** To achieve a relative axis between the smartphone and the cup rim, the marker probe is placed along the rim for a few seconds. **B** Smartphone screen when the cup rim plane is registered. The marker pattern is highlighted when the smartphone recognizes the marker

Before surgery, the smartphone is placed into a sterilized plastic bag. Before cup insertion, the smartphone is attached to the fixation device, which is rigidly clamped to the cup holder. The application procedure involves three steps: cup registration (step 1), anterior superior iliac spine (ASIS) registration (step 2), and target hunting (step 3). During step 1, the cup axis relative to the smartphone is measured by placing the flat aspect of the marker probe body along the cup rim and then measuring the marker probe (Fig. 3).

**Fig. 4:**
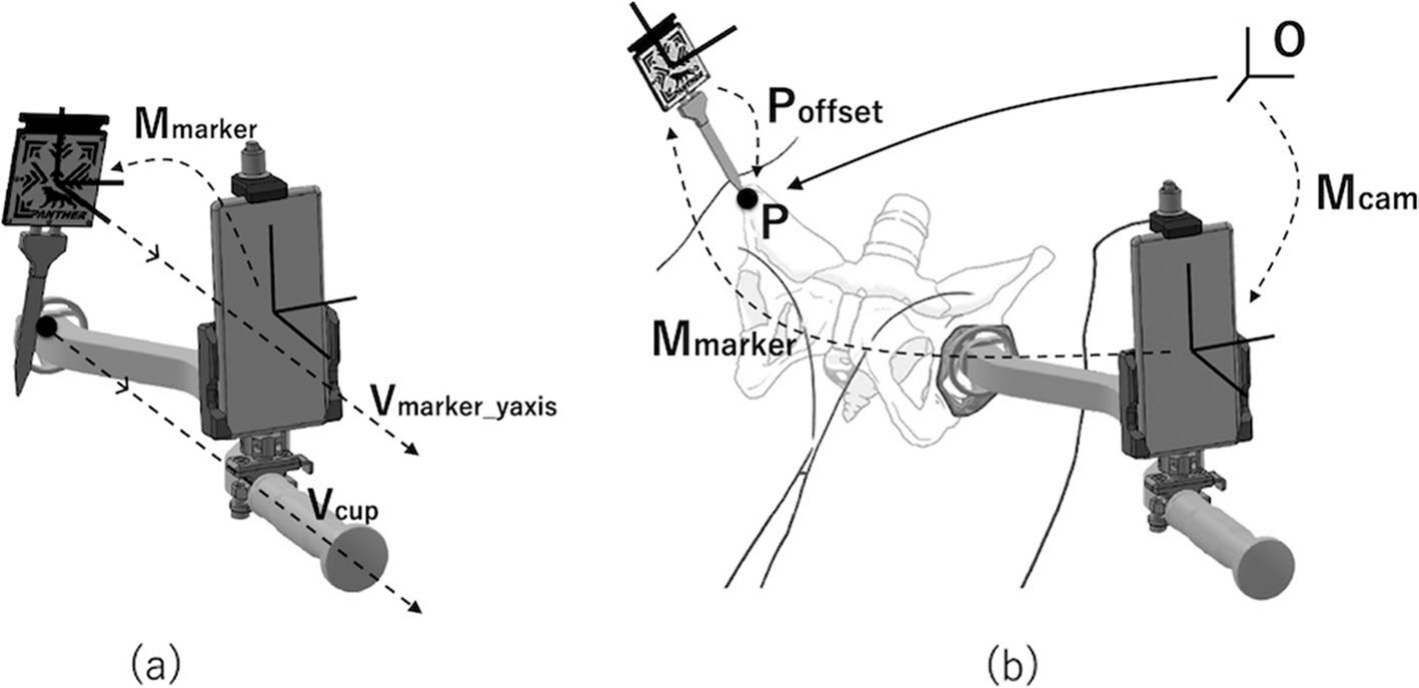
Overview of methodology steps 1 and 2. **a** Method used to calculate the cup axis relative to the smartphone. **b** Method used to calculate the probe tip position in the world coordinate system

In this case, the relative unit vector ${V}_{cup}$ of the cup axis from the smartphone can be measured as the Y-axis of the marker transformation matrix ${M}_{marker}$ (Fig. 4a). During step 2, the position of the left and right ASIS, palpable through the skin and soft tissue, is provided to the application by measuring the tip of the marker probe. Let $O$ be the world coordinate system, ${M}_{cam}$ the transformation matrix from $O$ to the camera, ${M}_{marker}$ the transformation matrix from camera to marker center, and ${P}_{offset}$ the offset from the marker center to the probe tip (Fig. 4b).

The probe tip position $P$ can be calculated using the following equation.

$$P={M}_{cam}\times {M}_{marker}\times {P}_{offset}$$

**Fig. 5:**
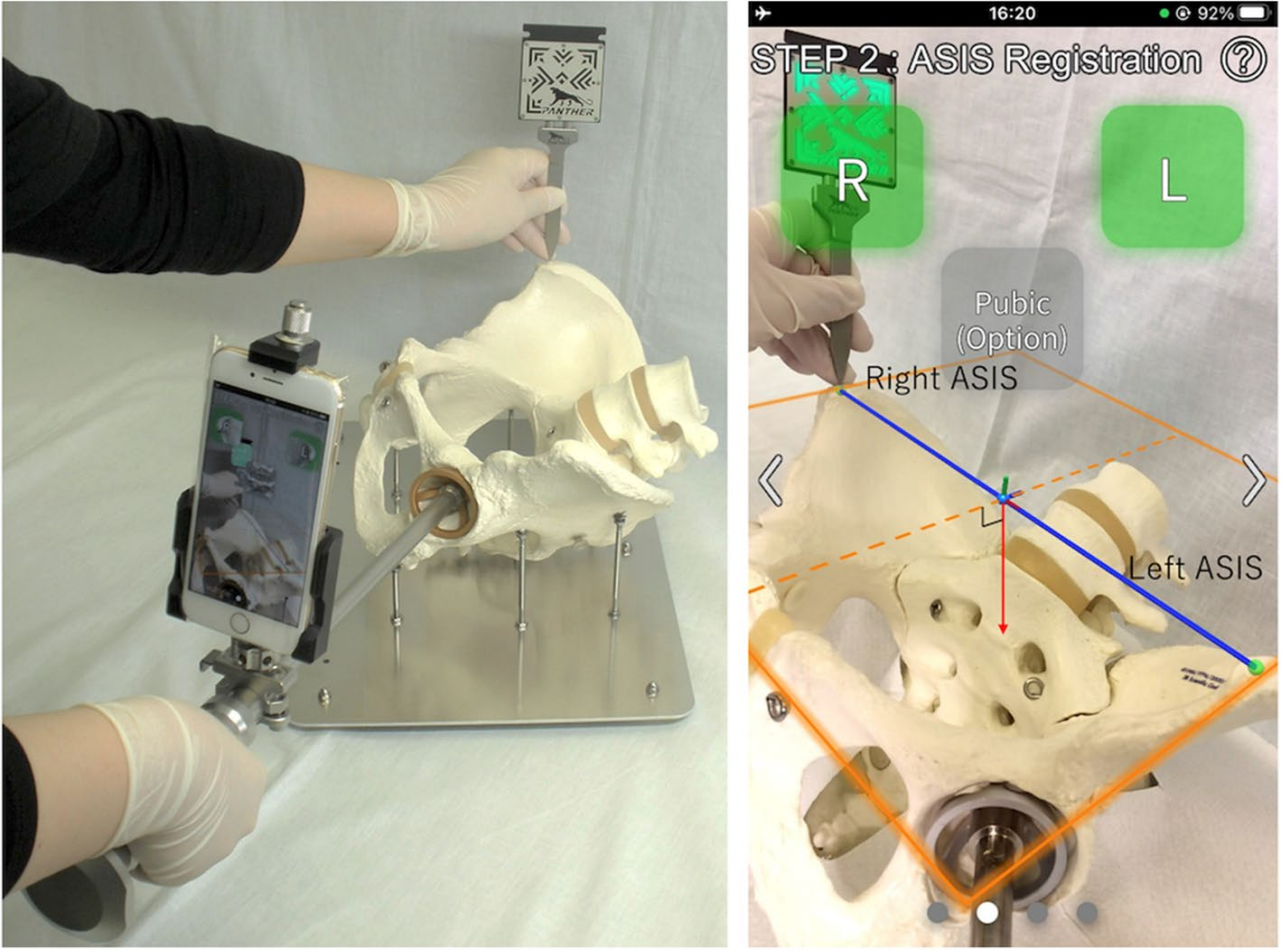
Anterior superior iliac spine (ASIS) registration procedure—step 2. **a** ASIS registration is performed immediately before the cup is placed on the acetabulum. **b** Smartphone screenshot when ASIS registration is finished. The smartphone can recognize the functional pelvic plane (FPP) (orange line) using the geographical information of the left and right ASISs. The sagittal vector of the FPP (dotted line) is defined as perpendicular to the gravitational vector (red line)

**Fig. 6:**
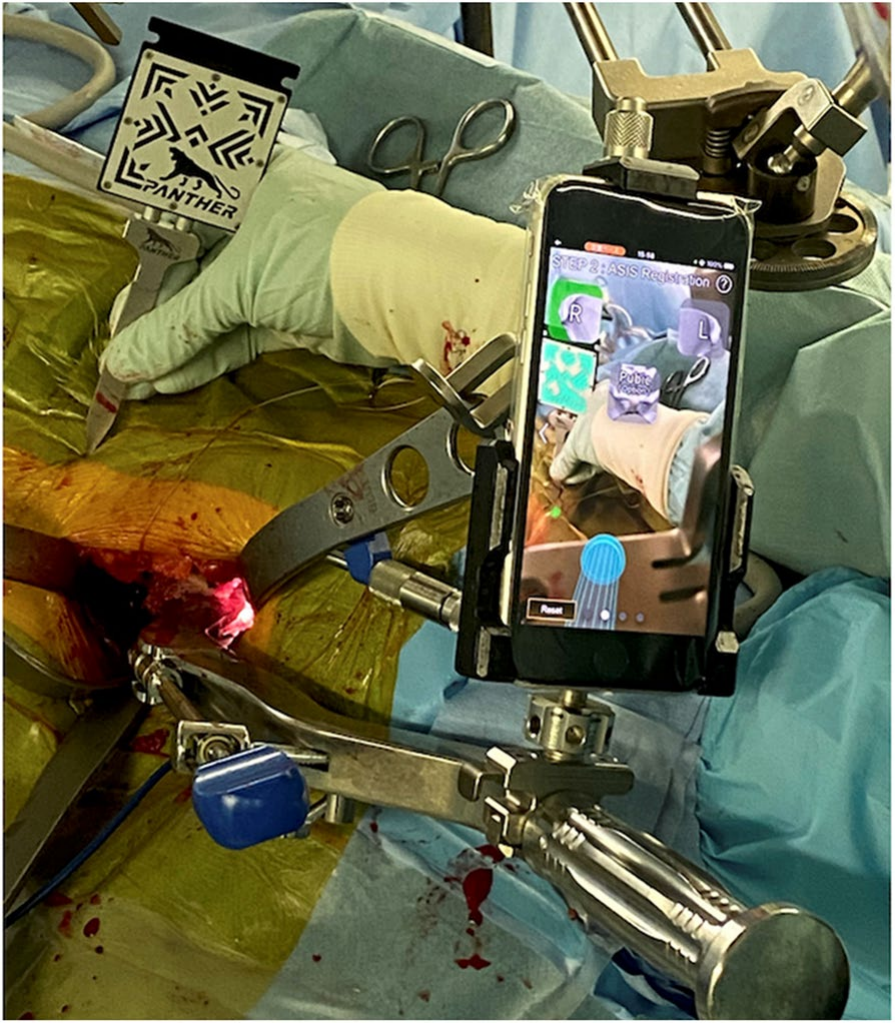
Intraoperative procedure of step 2. Surgeon pointing to the right anterior superior iliac spine (ASIS) with the marker probe; the smartphone recognizes the marker pattern, which is illuminated on the smartphone screen. The cup figure achieved in step 1 is also illuminated at the bottom of the screen

${M}_{cam}$ is calculated using position-tracking technology that can correct the smartphone’s location using visual information obtained from its camera, even if the smartphone is moved. ${M}_{marker}$ is calculated using AR technology that detects the relative position and orientation from the camera to the marker based on the marker pattern in the camera image. The FPP defines cup inclination and anteversion and is calculated from the left and right ASIS positions and gravity vector; it is then displayed using AR technology (Figs. 5 and 6).

**Fig. 7:**
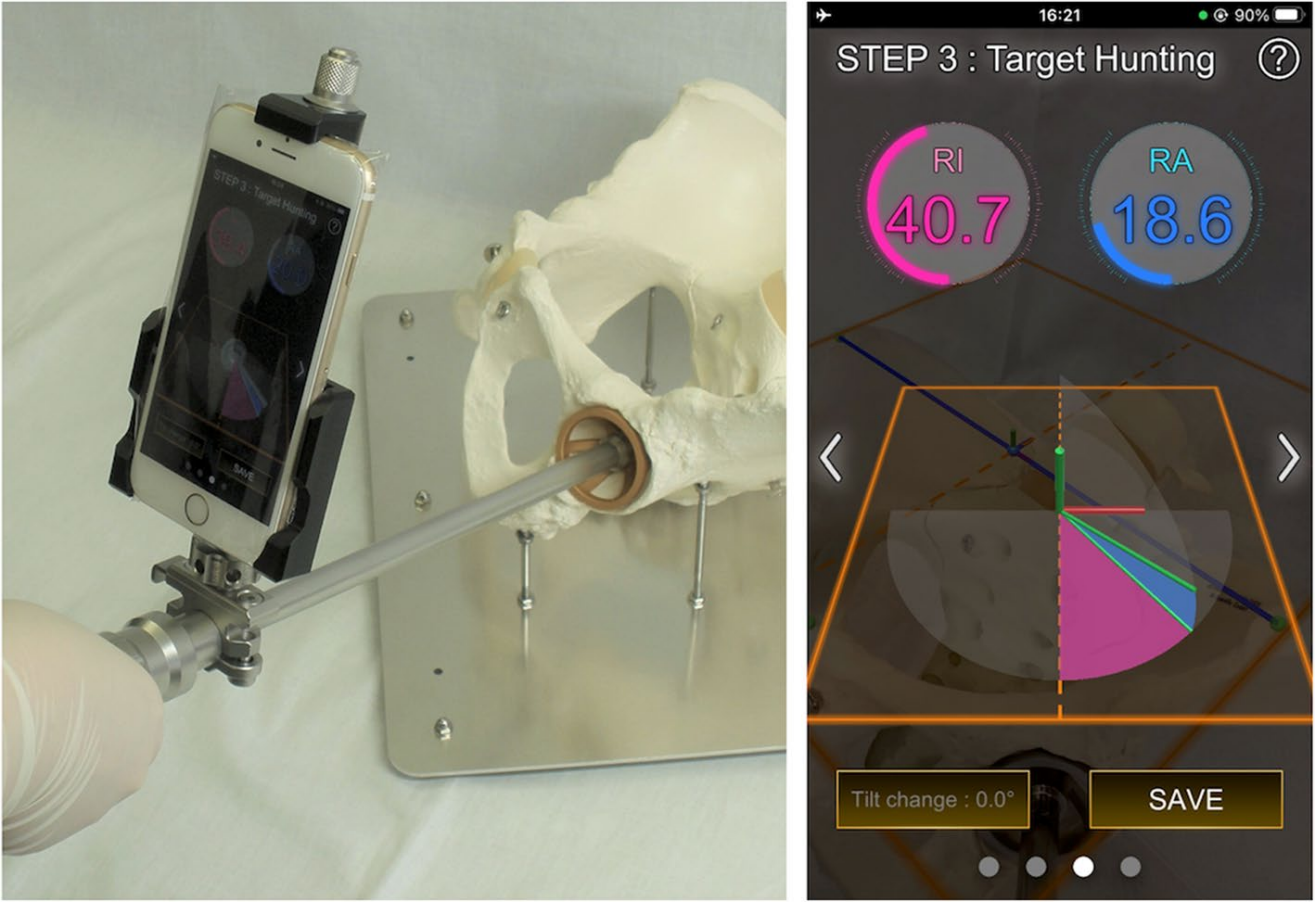
Target-hunting procedure of step 3. **a** The cup alignment shown on the smartphone changes in real time as the surgeon moves the cup holder. **b** Smartphone screenshot during step 3. The cup alignment is theoretically correct unless the pelvis moves during the procedure

Finally, during step 3, the smartphone attached to the cup holder shows the surgeon’s real-time cup alignment (Fig. 7).

The application calculates and shows real-time cup alignment using two factors: the real-time cup axis—calculated from the relative cup axis achieved during step 1—and real-time smartphone orientation, and the FPP achieved during step 2. The cup-alignment value is correct, provided the pelvis does not move during step 3. Surgeons can adjust the cup by checking the real-time cup alignment. They can also verify the final cup alignment by attaching a smartphone to the cup holder. Step 2 can be repeated if the pelvis moves during step 3.

### Outcomes

The primary outcome was the absolute value of the difference between the cup alignment—measured intraoperatively—and the postoperative cup alignment (absolute estimate error). Radiographic definitions were used for the intra- and post-operative measurements [14]. In the navigation group, radiographic inclination and anteversion were measured using a navigation system after cup fixation. In the control group, alignment values were measured using a conventional goniometer after cup fixation. Postoperative radiographic cup inclination and anteversion on the first anteroposterior postoperative radiograph were manually measured by the first author (R.T.) using the iRad-OT system (INFOCOM Corp., Tokyo, Japan). To evaluate inter-observer reliability, we ensured that the radiographic inclination and anteversion of 20 randomly selected patients were measured by a second observer (N.W.) using the iRad-OT system. To determine intra-observer reliability, the first author measured 30 patients 2 months after the first measurement. An absolute value of ≥ 5° and 10° was considered an outlier. The number of outliers was estimated for both groups.

### Statistical and power analyses

Welch’s t-test and chi-squared test were used to compare all factors between the navigation and control groups. Intra- and inter-observer reliabilities for the postoperative cup-alignment measurements were analyzed using intra-class correlation coefficients. Statistical significance was defined as a two-sided *P* < 0.05. A statistical power analysis showed that 30 patients in each group would be sufficient to detect a 2.0° difference in the absolute estimate error between groups, with a power of 80% and a type-I error rate of 5%. For the sample size calculation, based on the results of other studies, a standard deviation (SD) of 3.0° was used for the absolute target error [15]. All statistical analyses were performed using G*Power, Version 3.1.9.2 (Düsseldorf University, Düsseldorf, Germany) and JMP for Mac, version 13.0.0 (SAS Institute, Cary, NC, USA).

## Results

Patient background and surgical characteristics are shown in Table 1.

**Table 1:** Background and surgical characteristics

Variable	Navigation (*n* = 30)	Control (*n* = 30)	*P*-value
Age (years)	67.5 ± 10.7	64.4 ± 10.4	0.29
Sex (female/male)	27/3	25/5	0.52
Side (right/left)	14/16	10/20	0.29
Body mass index (kg/m^2^)	22.9 ± 2.8	23.9 ± 3.8	0.23
Diagnosis			0.90
Primary osteoarthritis	3	2	
Dysplasia of the hip	25	26	
Osteonecrosis	2	2	
Operative duration (min)	84.5 ± 20.6	86.5 ± 27.6	0.79
Intraoperative blood loss (mL)	251.9 ± 98.5	249.7 ± 190.3	0.66

Values are presented as mean ± standard deviation

The mean absolute estimate errors of cup inclination in the navigation and control groups were 2.9° (SD, ± 2.8°) and 3.1° (SD, ± 1.7°), respectively (*P* = 0.67). Conversely, those of cup anteversion were 3.4° (SD, ± 3.0°) and 5.4° (SD, ± 3.6°), respectively (*P* = 0.018) (Table 2).

**Table 2:** Absolute target error

	**Navigation** **(*n* = 30)**	**Control** **(*n* = 30)**	**Difference** **(95% CI)**	***P*-value**
Radiographic inclination (°)	2.9 ± 2.8 (0.1–9.4)	3.2 ± 1.7 (0.1–6.5)	0.3 (− 0.9 to 1.4)	0.67
Radiographic anteversion (°)	3.4 ± 3.0 (0.4–9.3)	5.4 ± 3.6 (0.1–15.4)	2.0 (0.4–3.8)	0.018

Values are presented as mean ± standard deviation (minimum–maximum); CI, confidence interval

**Fig. 8:**
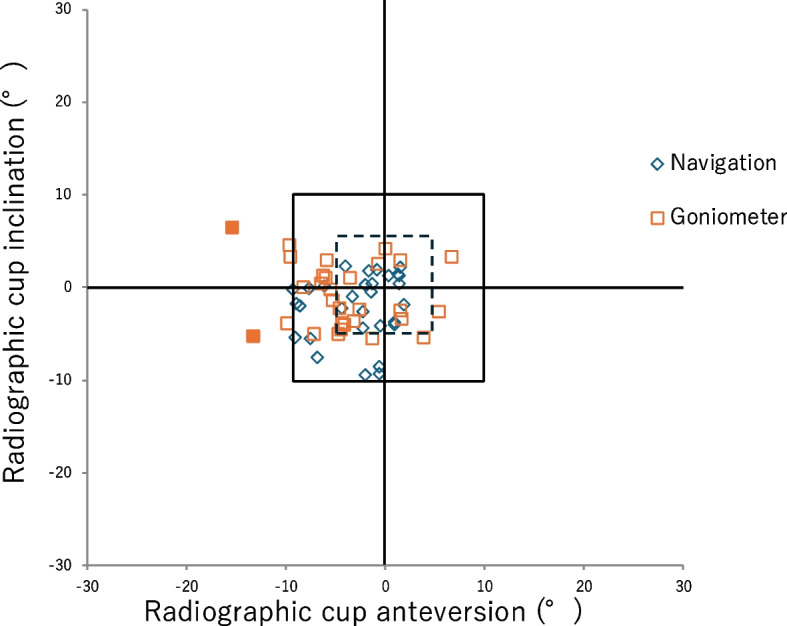
Scatter diagram of the target error. The case with a positive target error indicates that the postoperative angle is larger than the target angle. The dashed- and solid-line square boxes reflect the defined outlier line (absolute target error ≥ 5° and 10°). The filled marker represents the outlier

Eleven outliers of ≥ 5° were found in the navigation group and 18 in the control group (*P* = 0.07). No outliers for ≥ 10° were found in the navigation group, while two were found in the control group (*P* = 0.52) (Fig. 8).

**Fig. 9:**
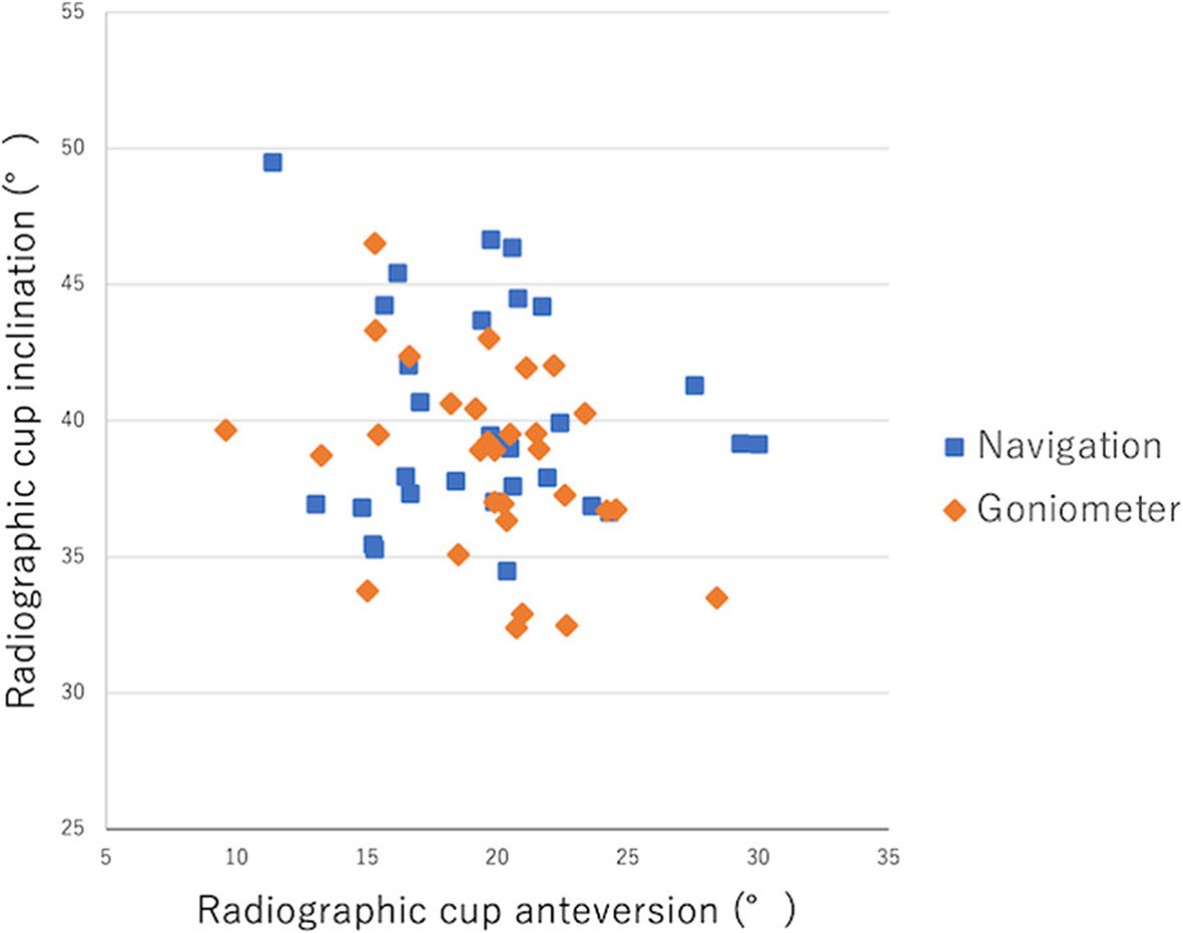
Scatter diagram of the radiographic inclination and anteversion. The radiographic inclination and anteversion of the navigation and control groups are shown

Cup-alignment data of both groups are shown in Table 3, and the related scattergram is shown in Fig. 9.

**Table 3:** Cup alignment

	**Navigation** **(*n* = 30)**	**Control** **(*n* = 30)**	**Difference** **(95% CI)**	***P*-value**
Radiographic inclination (°)	40.1 ± 3.9 (34.5–49.7)	38.6 ± 3.5 (32.4–46.5)	1.5 (− 0.3 to 3.5)	0.1
Radiographic anteversion (°)	19.6 ± 4.4 (13.1–30.0)	19.7 ± 3.7 (9.6–28.4)	0.01 (− 2.1 to 2.1)	0.99

Values are presented as mean ± standard deviation (minimum–maximum)

No significant differences were observed between groups regarding patient background characteristics (Table 1). The intra-class correlation coefficients showed that intra- and inter-observer reliabilities for cup inclination and anteversion—measured using the iRad-OT system—were good to excellent (0.90 and 0.79, and 0.91 and 0.73, respectively).

## Discussion

The novel non-invasive AR-based navigation system described herein enabled surgeons to achieve better cup anteversion accuracy than that of the conventional goniometer. Although the difference was not statistically significant, the navigation group showed a tendency toward fewer outliers. To our knowledge, this is the first study that evaluated the accuracy of cup positioning of this new navigation system. The greatest advantage of this navigation system is its non-invasiveness. Although AR technology has already been used in other navigation systems, to our knowledge, this is the first portable navigation system that does not require an invasive procedure [7–9]. This non-invasive feature can reduce the risk of complications caused by invasive procedures included in other navigation systems and is considered to possess excellent cost-effectiveness, especially compared to CT-based navigation [16]. Moreover, while many other navigation systems face challenges in continued intraoperative use due to pin loosening, our system eliminates this concern. Although the accuracy and precision of this system are lower than those of other navigation systems, this report may encourage the development of such non-invasive devices for THA in the future. Just as THA itself has evolved toward less invasive approaches, we believe that navigation systems will also continue to develop in a similarly non-invasive direction.

No significant between-group difference was observed regarding cup inclination in both groups, possibly because cup inclination was measured by referencing both ASIS locations, while cup anteversion was not measured using bone landmarks (Fig. 1). Therefore, the differences in measurement might be caused by differences in accuracy between inclination and anteversion.

Some authors have reported the cup-positioning accuracy provided by other portable navigation systems [4, 5, 16–18]. Ogawa et al. reported the absolute error of the AR navigation system. The absolute values of radiographic inclination and anteversion were 1.9° and 2.8°, respectively. Their accuracy is superior to that of this study. The non-invasive feature of the navigation system is considered to be the reason for the slightly inferior accuracy of cup positioning than the other AR navigation systems. The other limitation of this navigation system is that it can only be used for THA in the supine position. The increase in accuracy of cup positioning for lateral position should rely on other navigation systems currently.

This study has several limitations. First, it was a single-center study. Second, the accuracy of this system was not directly compared with that of other navigation systems. The influence of this navigation system on important factors—including cup position, offset, and leg length—was not evaluated. Furthermore, although we found significantly better accuracy of radiographic anteversion in the navigation group, it remains unclear whether this 2.2° difference translates into improved clinical outcomes compared to cases without navigation. However, recent studies have shown that the so-called “safe zone” for cup positioning has become narrower, and even a few degrees of deviation may be critical, particularly in patients with reduced spinopelvic mobility [1, 4]. Therefore, the possibility that an accuracy deviation of approximately 2° may affect clinical outcomes cannot be entirely excluded. Third, the relatively small sample size prevented an evaluation of how patient characteristics (especially BMI, height, and osteoarthritis grade) influence this system’s accuracy. In cases in which palpation of the ASIS is difficult due to obesity, the accuracy of the system may decrease [17]. In our study, this factor may have contributed to the lower accuracy observed in some cases. This study included cases in which it was intraoperatively difficult to palpate the ASIS, highlighting the need for a similar study focusing specifically on patients with high BMI in the future. Further investigation is warranted. Fourth, the supine position during surgery was used as FPP. However, pelvic tilt during general anesthesia may differ from that during the awake state. CT-based navigation and robotic systems can overcome these issues [19]. Fifth, the pelvic orientation in the supine position during surgery may differ from that in the postoperative anteroposterior radiographs. Such differences could potentially influence the radiographic evaluation of cup positioning, especially the anteversion angle.

## Conclusions

This new non-invasive AR-based navigation system showed superior accuracy of cup anteversion compared to a conventional goniometer. The non-invasiveness of this system may help promote the development of less invasive navigation systems in the future.

## Data Availability

No datasets were generated or analysed during the current study.

## Abbreviations

AR: Augmented reality
ASIS: Anterior superior iliac spine
BMI: Body mass index
FPP: Functional pelvic plane
THA: Total hip arthroplasty
CT: Computed tomography
